# Methodology for soil respirometric assays: Step by step and guidelines to measure fluxes of trace gases using microcosms

**DOI:** 10.1016/j.mex.2018.06.008

**Published:** 2018-06-19

**Authors:** Leonardo M. Pitombo, Juliana C. Ramos, Helio D. Quevedo, Karina P. do Carmo, Jane M.F. Paiva, Elisabete A. Pereira, Janaina B. do Carmo

**Affiliations:** a Federal University of São Carlos (UFSCar) – Department of Environmental Sciences, Rod. João Leme dos Santos Km 110, 18052-780, Sorocaba, SP, Brazil; b Federal University of São Carlos (UFSCar) – Graduate Program of Biotechnology and Environmental Monitoring, Rod. João Leme dos Santos Km 110, 18052-780, Sorocaba, SP, Brazil; c Federal University of São Carlos (UFSCar) – Graduate Program of Planning and Use of Renewable Resources, Rod. João Leme dos Santos Km 110, 18052-780, Sorocaba, SP, Brazil; d Federal University of São Carlos (UFSCar) Graduate Program of Materials Science, and Department of Production Engineering, Rod. João Leme dos Santos Km 110, 18052-780, Sorocabas, SP, Brazil

**Keywords:** Respirometry, Carbon dioxide, Methane, Mineralization, Nitrous oxide, Respiration

## Abstract

This methodology is proposed to measure the fluxes of trace gases among microcosms and the atmosphere. As microcosm respiration we include both aerobic and anaerobic respiration, which may results in CO_2_, CH_4_, NO, N_2_O, N_2_, H_2_S and H_2_ fluxes. Its applicability includes the assessment of products biodegradability and toxicity, the effect of treatments and products on greenhouse gases fluxes, and the mineralization of organic fertilizers. A step by step procedure; the complementary parameters and good practices that might be taken into account to perform a microcosm experiment; and the tools nowadays available that could be useful in this respirometric methodology are presented. We included a spreadsheet with calculus examples. Samples were taken at 1; 30; 60 and 90 min after closing the microcosms to determine the gases fluxes. The dilution effect was negligible, as we present. Besides CO_2_, we have successfully quantified the fluxes of CH_4_ and N_2_O from the microcosms in a broad range of concentrations. This method is useful in technical and scientific studies, for instances to test new products and improve the understanding of microbial processes, respectively.

• Simple materials are required to set up the microcosm.
• Examples of (pre) treatments are given regarding water availability, fertilizer doses, pH adjustment and nutrients amendments.
• The method was suitable to directly measure multiple trace gases fluxes, either produced or consumed during microcosm respiration.

## Graphical abstract

**Figure:**
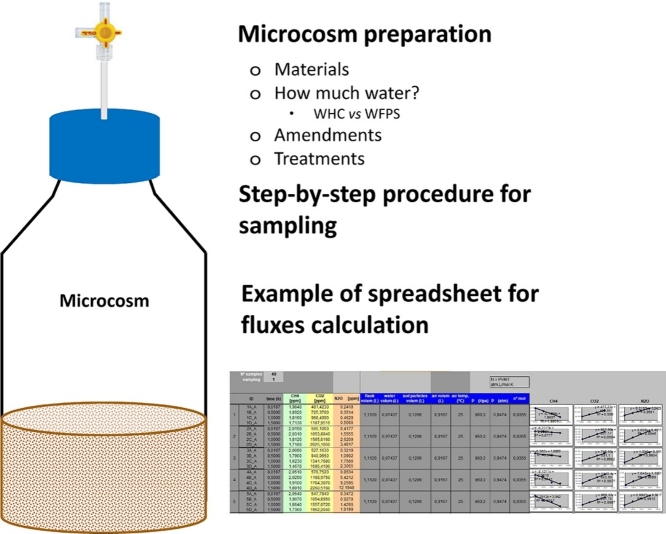

## Method details

Respirometry studies are useful methods applied to a broad range of technical and scientific areas. Soil respiration, for instances, is an indicator of microbial activity which is responsible to toxic chemicals [1] and amendment of limiting nutrients such as nitrogen [2]. One of the most traditional methods used to assess the mineralization of organic wastes in soil is the Bartha’s respirometric assay [1]. This is a titrimetric method which measures the CO_2_ trapped in a strong alkali after carbon mineralization by the microbial community present in the system. Its use includes the assessment of the complete biodegradation of petroleum wastes, general compounds and organic fertilizers in soil such as sewage sludge [[3], [4], [5]]. The kinetics of petroleum wastes mineralization indicates the capacity of the soil in metabolizes the organic amended materials; and the mineralization of fertilizers is a powerful indicator of nutrients release from the amended fertilizer once other compounds besides C are also mineralized.

Some adaptions to the Bartha’s respirometric assay were proposed (Table 1), such as the one that considers the changes in the electrical conductivity of the alkali after reacting with CO_2_ [6] or considers both the CO_2_ release and O_2_ consumption [7]. Nevertheless, these are indirect methods in which C mineralization is measured by the reaction of CO_2_ with the alkali or by the O_2_ consumption. Now, we present a detailed methodology that enables more than the direct quantification of compounds mineralization in microcosms. This methodology is proposed to measure the fluxes of gases among microcosms and the atmosphere. Its applications include the determination of the effects of different fertilizers on the microcosm respiration; and the determination of the degradability of organic compounds. As microcosm respiration we mention both aerobic and anaerobic respiration, which may results in CO_2_, CH_4_, NO, N_2_O, N_2_, H_2_S and H_2_ fluxes. Quantification of greenhouse gas fluxes among interfaces is of importance to assess the impact of treatments and new products on the atmosphere. Soils are the only biological sink of CH_4_. Consumption of trace gases and C storage are examples of ecosystem services provided by soils and their relevance for climatic regulation still need to be better studied. Hydrogen dynamics might be an important ecological indicator [8] and the screening of H_2_ producing organisms is of biotechnological interest due to its energetic potential [9]. Hydrogen sulfide is produced under anoxic conditions [10]. This methodology might also be used to the assessment of volatiles produced by the microcosm under study. The study of volatile compounds used as communicative way among organisms is a promising topic [11]. Volatiles produced by fungi were identified using a microcosm similar to the one we are describing [12] but measure their fluxes needs to be validated yet.

**Table 1:** Possible advantages and drawbacks attributed to the reference and alternative respirometric methods.

parameters	Bartha’s [1]	Conductimetric [6]	Manometric [7]	This study
target	CO_2_	CO_2_	O_2_ + CO_2_	CO_2_, CH_4_, N_2_O and others^a^
marker	CO_3_^−2^	CO_3_^−2^	pressure	direct
analytical range	restricted	restricted	wide^b^	wide^b^
interferers	unknown	unknown	volatiles	CO_2_ for N_2_O^c^
analytical calibration	*in house*	*in house*	manufacturer	*in house*
analyst-linked error	high^d^	low	low	medium^e^
marketed	no	no	yes	no
technical use^f^	widespread	unknown	widespread	potential
scientific use	widespread	yes	yes	potential
data scale	period	period	up to real time^g^	time point

a The use to measure other gases fluxes needs to be tested.

b Depends on the analytical device sensibility.

c As presented in material and methods.

d Solutions standardization, titration, trap solution handling.

e Schematic sampling.

f e.g. CETESB [3], OECD [5] and the joint publication of the *Standard Methods for the Examination of Water and Wastewater*.

g There are commercial versions with data loggers embedded.

In comparison with the indirect methods used as standards [3,5], the methodology herein described is more versatile once it is suitable to measure many gases in the same sample besides CO_2_. Most of the solutions used in the indirect methods such as the Bartha’s respirometry assay are not primary standards, which request daily standardization. Besides laborious, these procedures add error during each step of manipulation, consume large amounts of chemicals and, consequently, large amounts of lab waste are produced. On the other hand, gas chromatography methods are becoming popular, are waste-free and mostly important, present high sensibility and reproducibility levels. The indirect methods require the microcosms are kept closed during long time to assure sensibility during the titration procedure. However, these methods don’t include any procedure to check for microcosm atmospheric saturation and gas release or uptake kinetics during this time. In our case, we assume if there is linearity in the curve of gas concentration between sampling events there is no factor changing microcosm respiration kinetics. Additionally, we claim more attention should be given to complementary parameters, mainly regarding water availability. For instances, a sandy upland soil might not be able to reach 70% of its water-filled pore space (WFPS), while 70% of water holding capacity (WHC) is reasonable. When is it adequate to keep the microcosm closed? In this work, we describe a guideline, including (I) a step by step procedure; (II) the complementary parameters and good practices that might be taken into account to perform a microcosm and (III) the tools nowadays available that could be useful in this respirometric methodology.

## Materials and methods

### Microcosms

**Fig. 1:**
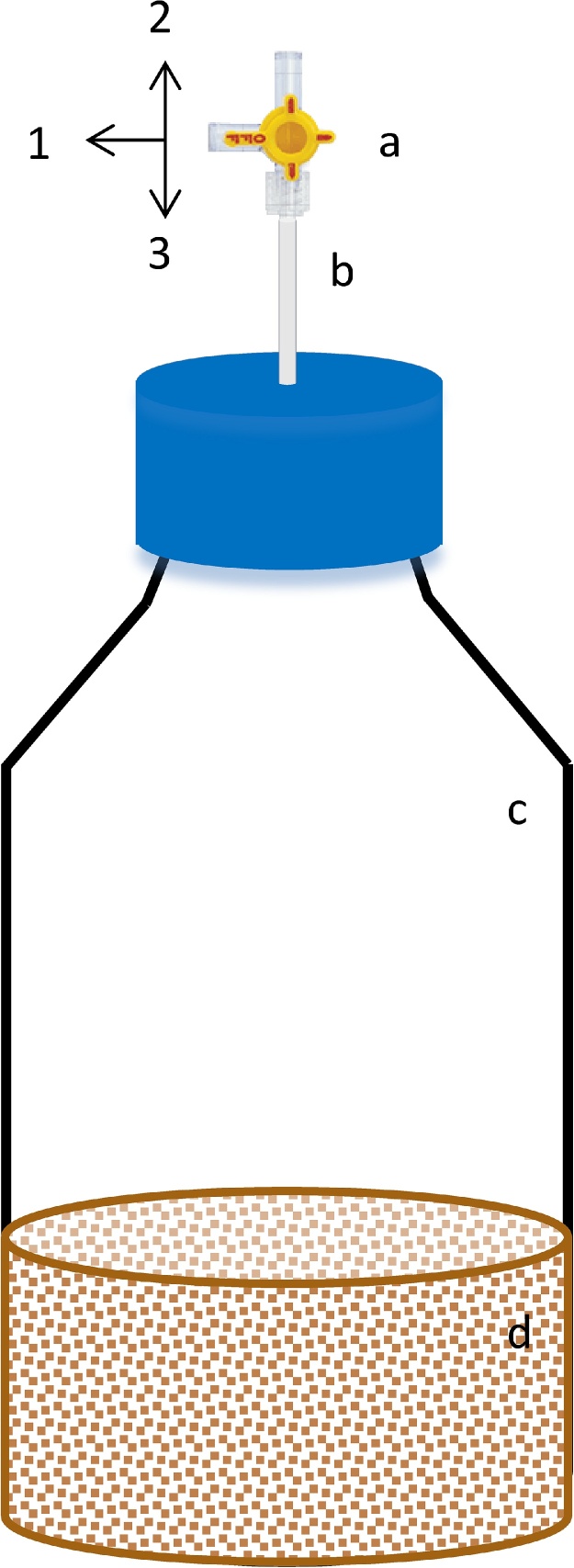
A non-scaled illustration representing an incubation flask. In (**a)** a 4-way stopcock valve with 2 female luer locks and male luer with spin lock (QOSINA, NY, USA); in (**b**) a Bev-A-Line^®^ tube (1/8″ ID × 1/4″ OD × 1/16″ Wall Bev-A-Line IV Tubing) inserted in the reagent flask tip used to couple the flask **(c)** and the valve; and in **(d)** the incubated soil. The cap was looped using a drill and the tube could be perfectly fitted without any additional treatment.

Incubations were performed in reagent-type flasks for 1 L. The exactly flask air volume was determined by mass difference between the empty and filled flask with deionized water, which has known density. We adapted the flask caps with a sampling system as described on Fig. 1. Syringes used for sampling (Becton, Dickinson and Co., Franklin Lakes, NJ, USA) were also fitted with a 4-way stopcock valve added of non-vented tips at position 1 (QOSINA, NY, USA; Fig. 1). The flasks were only closed during gas sampling. If the flasks are closed for long terms they change completely the gas kinetics release and might cause atmosphere saturation.

Soils were sieved through a 2 mm mesh screen for homogenization. The standard amount of soil incubated was 300 mL (bulk density). Particles density was obtained to calculate the air volume inside the microcosm. Water availability was adjusted either by soil WHC or WFPS, depending on the assay purpose. It is mandatory to consider the soil residual water content in the calculus. Pre-incubations were performed to stabilize gas fluxes prior treatments were applied. Eventually, pre-treatments were performed during the pre-incubations, such as to adjust the soil pH or perform micronutrients amendment as described in the sequence. The microcosms were kept on an acclimatized room at 25 °C ± 2 °C or at an ultra-thermostatic bath at same temperature. We don´t recommend closed incubators which might either concentrate trace gases along the incubation procedure or become an anoxic environment.

### Treatments applied

Here we present sets of (pre) treatments performed with different purposes:

#### Soil pre-incubation

Independently of the incubation aim, we pre-incubated the soil till the microcosm reached its basal respiration. For our conditions, one week of pre-incubation was enough to stabilize the gases fluxes. Soil moisture was kept at a level in which the main treatment would be applied in the sequence. For instances, when the main treatment was set to 70% of WHC, the pre-incubation was performed at 40% of WHC. Therefore, the fertilizers or other products would be diluted in enough water to be equally spread on the soil surface. Besides this mandatory pre-treatment, other pre-incubations were performed as described in the sequence.

#### pH adjustment prior main treatment

This pre-treatment might be necessary when reproducing an agricultural soil. For one of our experimental aims, the soil pH was adjusted to a pH often found in soils at commercial sugarcane fields (5.5–6.0). A neutralization curve was previously determined as described by CETESB [3] for respirometric assays. In summary, at least five different amounts of CaCO_3_ following an arithmetical progression were added to 20 g of soil in Erlenmeyer flasks for 125 mL in three replicates. Soil and CaCO_3_ were mixed and kept under 70% of WHC during 7 days. Afterwards, soil pH was determined in CaCl 0.01 mol L^−1^ at ratio 1:2.5 (w:v). For this soil, 740 mg kg soil^−1^ of CaCO_3_ was required to adjust the soil pH to the target range (5.5–6.0).

#### Micronutrient amendment prior main treatment

Micronutrients might be limiting for energy acquisition such as for CH_4_ oxidation [13,14] and N_2_O conversion to N_2_ [13,15]. For an experimental set we would like to test the effects of copper availability on the reduction of N_2_O to N_2_. Hence, we added during the pre-incubation step a source of copper (CuCl_2_) to reach the target micronutrient availability. We performed an adsorption curve following the same principles than the used for the pH neutralization curve. The extractor (DTPA) was the one recommended for tropical soils [16]. Copper was diluted in the biggest volume of water before the soil would reach 40% of its WHC and was added to the microcosm in the beginning of the pre-incubation. For this soil, 1.45 mg kg soil^−1^ was amended; thus it would have 1 mg kg soil^−1^ available.

#### Straw amendment prior main treatment

This pre-treatment might be interesting when reproducing a management practice. Usually, after the crop is harvest and prior the next sowing/fertilizer application, there is some crop residues deposited to or incorporated into the soil. We would like to assess the effects of sugarcane crop residues blanketing on the N_2_O emissions and CH_4_ consumption in one of our experimental set ups. Therefore, 6.362 g straw was deposited per flask for the straw treatments. This amount was established based on the flask area to simulate 10 Mg straw ha^−1^. In this particular experiment pre-incubations were a month long before the fertilizer was applied because it is approximately the period between sugarcane harvest and fertilizer application.

#### Fertilizer application

Assess the effects of fertilizers on soil respiration was the main purpose of our experimental sets. We tested both organic and inorganic fertilizers and the interaction among them. In our case, the amount of inorganic fertilizer applied was based on the concentration of N observed in the field [17] when the functional response in study (N_2_O release) was prominent. In this case, we assumed 100 mg N kg soil^−1^ would be adequate to test our hypotheses. It was necessary because fertilizer is applied in bands on the sugarcane field. In other works we have adopted other ways to calculate the amount of fertilizer to be added in the microcosm which resulted in similar N concentrations. For instances, when the fertilizer is spread in the field and the dose is 100 kg N ha^−1^, the amount to be added to the microcosm is also 100 mg N kg soil^−1^ taking into consideration the soil layer of 0–10 cm and soil density of 1 g cm^−3^. In citrus orchards the fertilizer dose is 50 kg N ha^−1^, but it is spread on half of the field area, which represents the trees canopy projection. Therefore, the calculus results in the same 100 mg N kg soil^−1^. The organic fertilizer (vinasse) amendment was based on the dose of 100 m^3^ ha^−1^ and the affected soil depth of 0–20 cm, which was equivalent to 49.4 mL kg^−1^ soil (15 mL flask) taking into consideration the soil density. It coincides with the calculus criterion used by Silva et al. [18] for soil incubations to estimate the net N mineralization of this kind of organic material.

#### Polymer biodegradability assay

We have used this respirometric assay to compare the degradability of starch polymers synthetized *in house*. These polymers were cut in squares (16 cm^2^) and inserted in mash bags. After the pre-incubation period, the mash bag with the polymer was inserted 1 cm deep in the microcosm. Microcosms were kept at 70% of the soil WHC during the incubation. The control treatment didn’t receive any polymer.

#### Artificial root exudates solution

When the aim was to simulate a soil-plant condition, the microcosms were daily amended with an artificial root exudates solution proposed by Baudoin et al. [19] without amino acids when N_2_O measurements were performed [20]. After the gas sampling, 5000 ug of C (per flask) was added to the soil to keep the soil basal respiration. The soil moisture was adjusted after C addition. Carbon supply is mainly necessary during long term incubations without any organic amendment.

#### Water availability

Water availability is a basic parameter for a respirometric assay but might also be considered a treatment variable. We recommend the use of WHC when simulating field conditions. Water-filled pore space might be indicated to the study of physiological processes since it is rather an indicator of oxygen availability than water availability. However, its applicability is more restricted since the range of WFPS goes from 0 to 100%, but higher values might not be possible in practice. For instances, WFPS is recommended to study nitrification and denitrification processes. However, if the respirometric assay is performed to test a fertilizer, the soil moisture would be adjusted to a condition that occurs in the field. In this case, WHC fits better to the experimental aim. For a biodegradability assay, WHC from 50 to 70% are usually within a good range that will not be limiting for the compounds mineralization [3]. We adjusted the water availability daily by weighting the microcosms.

### Destructive experiments and complementary parameters

Destructive experiments are useful to follow the degradation of compounds, monitor the consumption of substrate or production of metabolites and the microbial community driving these processes. We have used two approaches for the destructive assays: (I) assemble microcosms (Fig. 1) as much as needed to destroy along the experiment; or (II) assemble both the regular microcosms to perform the gas monitoring and microcosms in centrifuge tubes with proportional amount of material. All microcosms were kept under the same conditions, including the daily water restitution. This last ones might be feasible when the resources are either costly (e.g. isotope labeled materials) or limited (e.g. sample availability).

The time points to destroy microcosms might be determined either systematically prior the beginning of the experiment, or after a response observed (e.g. emission peak; log-lag-stationary phases). The complementary parameters which might be useful for the study include nutrient availability (e.g. inorganic N, soluble C, P, S, micronutrients), biomarkers (e.g. phospholipids; enzymes activity; amino acids; microbial biomass), pH, metabolites (S and N compounds; degraded molecules; secondary metabolites), the use of natural and enriched isotope tracing methods and the most cutting edge molecular techniques and meta-omics based in DNA, RNA and protein analyses.

### Step-by-step procedure

Gas sampling and storage: microcosms were fanned during 30 s with a paperboard before closing the flasks; the cap valve lock was kept in the position 3 prior the gas collection (Fig. 1); One minute after closing the microcosm, the cap and the syringe locks were switched to the position 1; three pumping movements were performed with the syringe; on the fourth pumping, 20 mL gas samples were collected; after gas sampling, both the valves at the syringe and flask cap were directed to the position 2; the cap valve was quickly returned to the position 3. This last step provides the gas pressure balance between the outer and internal environments and a typical deep breath noise is a sign that the system is well tight. Sampling procedure was repeated at 30; 60; 90 min after closing the microcosms. When analyzed *in house*, the gas samples were kept in the syringes and analyzed on the same day of sampling. When sent for analysis abroad, the samples were stored in 12 mL tight flasks (Exetainer™ p/n 039W). Samples storage might also be applied for either large number of samples or the use of autosampler devices.

#### Gas analyses

CO_2_, N_2_O and CH_4_ concentrations were determined by gas chromatography (GC 2014 Shimadzu, Kyoto, Japan). *In house*, N_2_O was determined with an electron capture detector (ECD) and CO_2_ and CH_4_ with a flame ionization detector. Because CO_2_ interferes on N_2_O detection by the ECD [21], the system was equipped with an a HayeSepTM N packed column (1.5 m, 80–100 mesh) for N_2_O separation. When sent abroad, the samples were analyzed with a ThermoScientific GasBench with a Precon gas concentration system interfaced to a ThermoScientific Delta V Plus isotope-ratio mass spectrometer at the UC Davis Stable Isotope Facility.

### Calculus

Fluxes and emissions calculation: gas and elemental fluxes were calculated according to the Eq. (1). A spreadsheet with the use of this equation is presented as Supplemental material.

(1) $$Flux=\;\frac{\frac{\Delta gas}{\Delta t}\;\times \;nº\;mols}{mass\;of\;\;material}\;\times M\;gas\;\times \frac{M\times n}{M\;gas}$$

were:

*Flux* is the elemental flux which is release as gas (e.g. C or N);

$\frac{\Delta gas}{\Delta t}$ is the slope of the linear regression of gas concentration *vs.* time (Supplemental spreadsheet columns “V” to “X”);

*nº mols* is the number of mols of the gas inside the microcosm (Supplemental spreadsheet column “N”);

*mass of material* refers to the microcosm matrix, which in our case was soil (Supplemental spreadsheet columns “Y” to “AA”, 0.303 kg as inserted in the cell equation);

*Mgas* is the molar mass of the quantified gas (e.g. CO_2_, N_2_O, CH_4_);

*M* is the elemental molar mass (e.g. 12 for C, 14 for N);

*n* is the number of atoms of the element in the gas (e.g. 2 N in N_2_O).

**Fig. 2:**
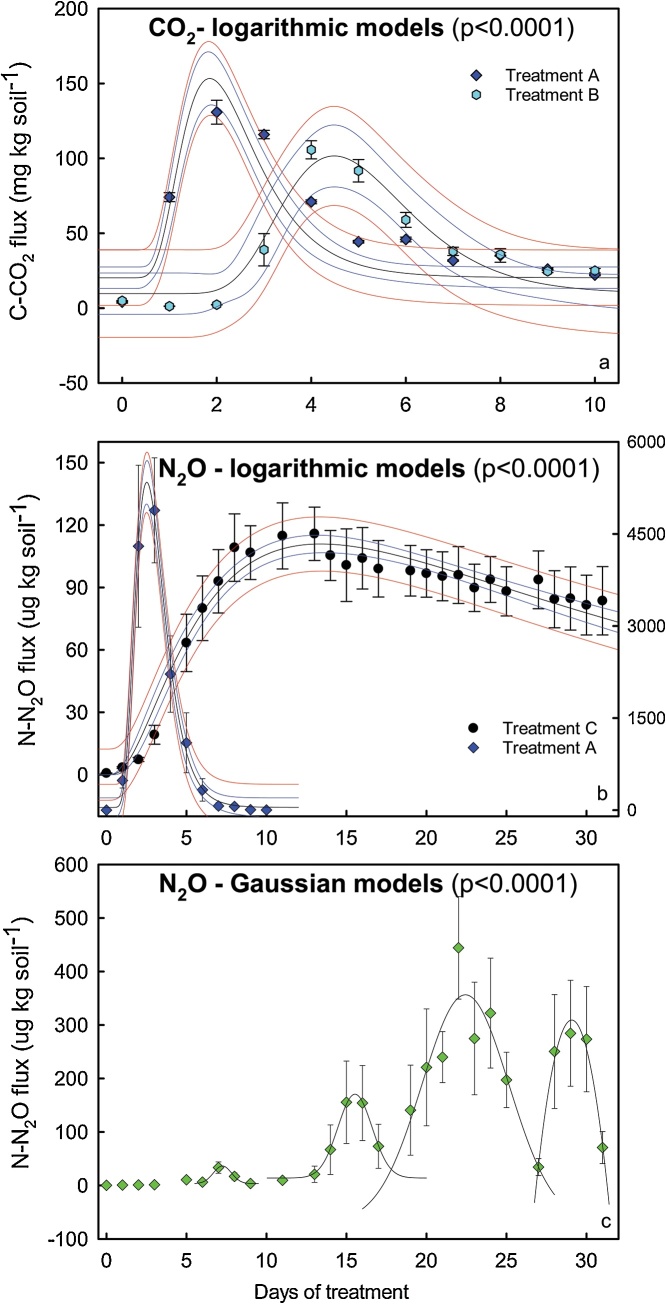
Models fitted to study CO_2_ (a) and N_2_O (b and c) fluxes from the microcosms. Blue lines indicate the 95% confidence interval while the red lines represent the 95% prediction interval. In “b”, the scale at the right corresponds to the “Treatment A” data.

A consistent quality control was adopted to remove possible outliers that could interfere on the flux calculation. Exceptionally, outliers might occur due to gas leakage or instrumental malfunction and they can be easily detected by visual data inspection ([22]; see Supplemental spreadsheet). Our quality control was based on the best correlation coefficient (r^2^) achieved after the removal of 1 time point data, when thought necessary. It was possible because we adopted a 4 time points system. Emissions were obtained by the integration of the curve of gas flux vs. time. There are different ways to perform this integration. We adopted the most used one, which is performing the linear interpolation of fluxes between sampling events. Another way is through the integration of the fitted model (Fig. 2), which might also be useful to predict the fluxes along the time.

## Method validation

### Sensibility and quality control

**Fig. 3:**
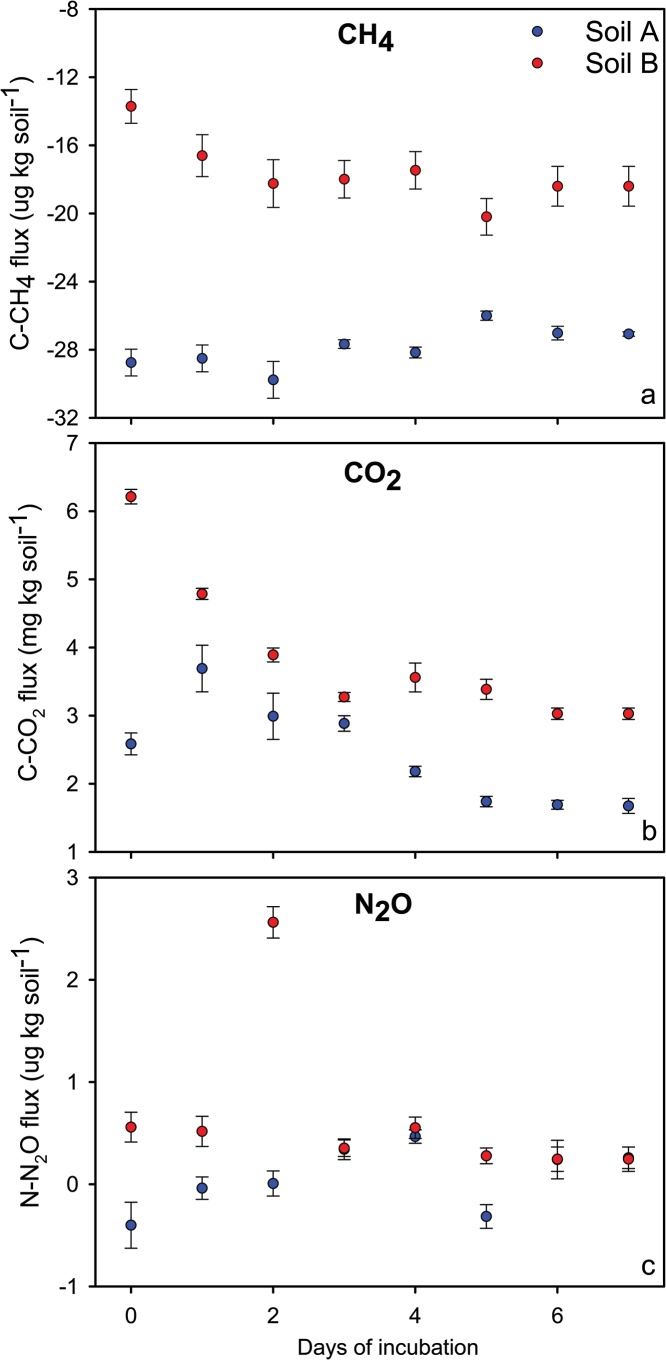
Fluxes of CH_4_ (a), CO_2_ (b) and N_2_O (c) during the pre-incubation period performed to provide the fluxes stabilization.

The parameter to define the sensibility of the method was the coefficient of linearity during the flux calculation. Additionally, the plots fluxes vs. time (i.e. emission plot) reproduced biological-type curves (e.g. Ricker: Fig. 2a, logarithmic: Fig. 2b, and Gaussian: Fig. 2c), including the basal respiration curve (Fig. 3). Fluxes in these plots presented a coefficient of linearity greater than 0.90. Therefore, a point that scape from this behavior suggests further examination for possible outliers in the flux calculation. Some authors include in their quality control a threshold for the r^2^ to consider the flux as significant or 0 (e.g. 0.90 [23]). We didn’t include this threshold. If after screening for outliers the flux data remained with a low r^2^, we kept the data in its raw form without interference in our conclusions in any circumstance because the flux was negligible (close to 0) or null.

**Fig. 4:**
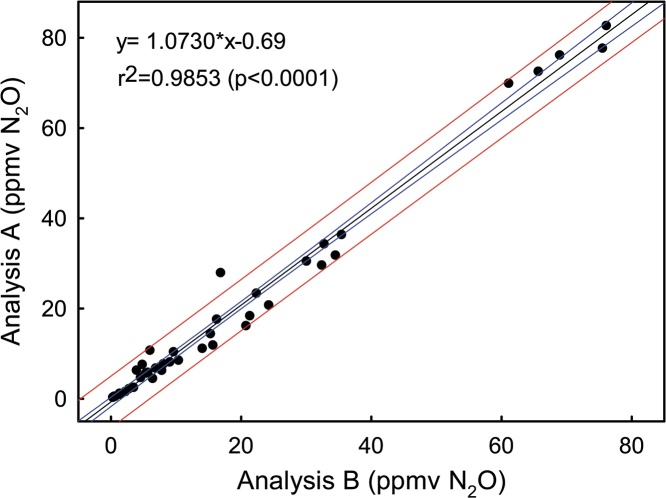
Samples analyzed *in house* (B) and overseas (A) collect after 60 min the same microcosm was closed with (B) and without (A) the previous sampling times. Blue lines indicate the 95% confidence interval while the red lines represent the 95% prediction interval.

Non-linear fluxes might be influenced either by analytical or kinetic issues. The analytical issues were previous mentioned in the material and methods section. The kinetic issues might be related with microcosm saturation or gas dilution during samplings. Dilution would also result in linear fluxes if it occurs at the same scale in between sampling events. Indeed, we sampled 20 mL and external gas goes into the microcosm to balance the microcosm pressure and dilution would be a concern. However, microcosms submitted to single (60 min; y axes Fig. 4) or multiple (1; 30; 60; and 90 min, x axes Fig. 4) gas samplings resulted in similar gas concentrations after 60 min the microcosms were closed (Fig. 4; angular coefficient close to 1). Then, we could disconsider the dilution effect. Additionally, any eventual dilution will occur at the same range in the Control or other treatment. Hence, the difference among these treatments will indicate the real treatment response. Another important observation is that there was no sample loss after shipping the vials overseas. We had previously tested the effects of shipping on the N_2_O concentration by receiving standards in vials from Europe and no changes were observed. Now we could verify that there was no gas release, since the method of analyses used in the data in the y axe (Fig. 4) quantifies all the gas in the vial, and not only gas concentration.

Adjustments on time of sampling might be requested depending of the analyte, intensity of microcosm respiration and detection limit of the device used for its quantification. If required, this adjustments need to be taken into consideration for the flux calculation, for instances, in the column “C” on the spreadsheet presented as Supplemental material.

### Pre-incubation

One week of pre-incubation was enough to bring the fluxes to its basal respiration (Fig. 3) for both the clay (soil A) and sandy soils (soil B). However, they stabilized at different levels. Soil A consumed around of 50% more CH_4_ than Soil B (Fig. 3a). While Soil A stabilized its C−CO_2_ emissions in 1.7 mg kg soil^−1^ day^−1^, Soil B released 3.0 mg kg soil^−1^ day^−1^ (Fig. 3b). There was no N_2_O flux from both soils at the end of the pre-incubation period (Fig. 3c).

## Additional information

### Biodegradation assay interpretation

**Fig. 5:**
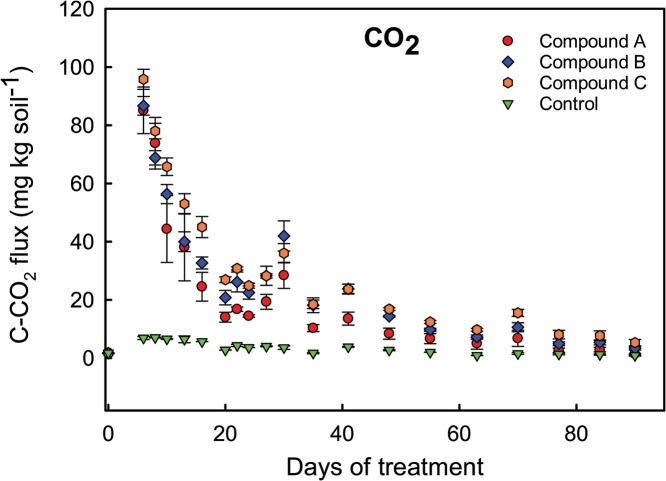
Fluxes of C−CO_2_ from the microcosm after addition of different biopolymers in the soil.

The results of the experiment performed to compare the biodegradability of different polymers are present on Fig. 5. Integrating the area of the plot we estimated the amount of C mineralized, which were: 79 ± 2; 544 ± 105; 584 ± 61; 677 ± 34 mg C per microcosm in the treatments Control and polymers A, B and C respectively. It is important to mention that such higher variance was expected since the mass of polymer in the microcosms were not the same because the material was added based on surficial area and not based on weight. Subtracting the result calculated for the Control from the emission calculated for each treatment we obtained the estimation of C released from the polymer. Since we knew the amount of C amended, we estimated that during the 90 days of incubations 36.4% ± 1.5; 34.7% ± 1.1 and 35.6% ± 2.0 of the polymers A, B and C were mineralized. Nevertheless, we would have a more precise estimation if the measurements had included the first days of incubation with the expected exponential growth phase, as observed in the data illustrated on Fig. 2.

It is important to differentiate compound degradation and compound mineralization. The last results in CO_2_ release while degradation is the transformation of the primary material to a less complex compound. In the experiment performed to evaluate polymers biodegradability we estimated around 35% of the compounds were mineralized. However, there was no polymer remaining in the mash bags after 90 days of incubation and the soil respiration was reduced to fluxes close to the Control treatment. Hence, we estimated the major fraction of the polymers was assimilated by the soil microbiota.

We propose the use of this respirometric assay to assess the biodegradability of compounds in the environment. However, it is needed the establishment of known compounds as standards to use them in a scale of biodegradability. The OECD [5] recommends five substances, from the less to the high biodegrability after 28 days of evaluation: tetrapropylene benzene sulphonate; iso-nonylphenol-(ethoxylate); coco-amide-propyl dimethylhydroxy sulphobetaine; di-iso-octylsulpho succinate; and hexadecyl-trimethyl ammonium chloride. Reference materials would also be useful in microcosms incubating other matrixes besides soil (e.g. inert materials as sand). However, inert matrixes request the use of inoculum [5] which might be the greater source of variability in respirometric assays [24].

### Range of fluxes successfully measured

**Fig. 6:**
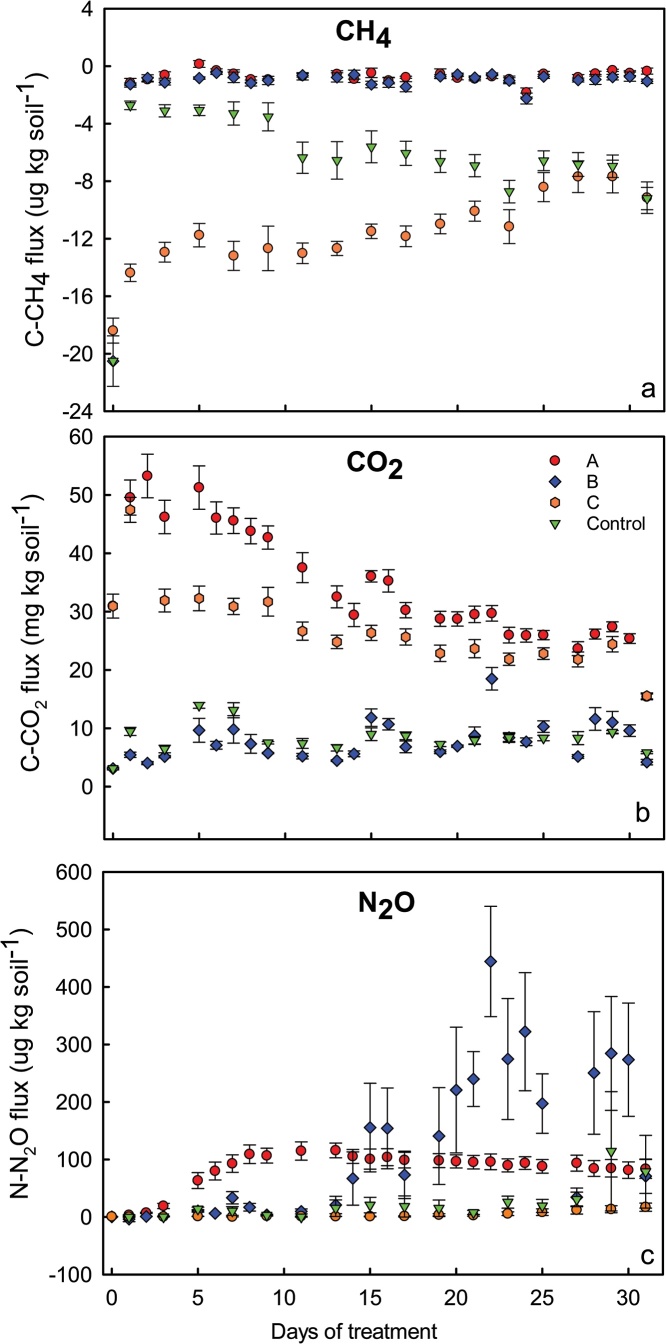
Fluxes of CH_4_ (a), CO_2_ (b) and N_2_O (c) after addition (A and B) of inorganic fertilizer and straw (A and C) on the soil. In (d) the illustration of covariance between fluxes of CO_2_ and N_2_O from the treatment B.

Results of an experiment performed to assess the effects of a type of nitrogen fertilizer on the GHG emissions from a specific soil are presented on the Fig. 6. The experiment was composed of four treatments: the Control treatment, A (Fertilizer + straw), B (fertilizer) and C (straw). The method sensibility was evidenced in this experiment. Even though the different gases fluxes were in such contrasting scales, we were able to clearly observe the effects of the treatments on the three trace gases analyzed. Methane maximum consumption fluxes measured for this experiment were about 20 ug C per kg soil and quickly decreased to near the null net flux in the other three treatments. Afterwards, the Control and “C” treatments converged to the same rate of consumption while the other treatments remained stable (Fig. 6a). Although CH_4_ oxidation was intense in this experiment, we have experienced mean consumption rates of up to 33 ug C per kg soil. In such conditions CH_4_ headspace concentrations in the microcosms decrease to around 0.6 ppm and the amount of C−CH_4_ oxidized was 10 ug after 90 min, which is the incubation time. The CH_4_ lowest concentration in the headspace (∼0.6 ppm) was close to the lower limit for detection of the used gas chromatograph device (0.1 ppm).

Results presented on Fig. 2a shows the C−CO_2_ fluxes after a liquid bioenergy byproduct (vinasse) was amended to the soil as fertilizer. This byproduct is rich in labile C [17,25] and comes with abundance of water, and then the mineralization occurs intensely. The fluxes reached 130 mg of C−CO_2_ per kg of soil. Under this intense C mineralization the correlation coefficient of the regressions performed to calculate the fluxes had minimum values of 0.999. These results show the versatility of the method, which has being either sensible to very small fluxes (Fig. 3a) or to intense C mineralization rates (Fig. 2a). In the field this byproduct application results in CO_2_ fluxes higher than when sewage sludge is applied [25,26], reinforcing the method versatility.

As result of labile C amendment together with inorganic N there was also an intense N_2_O release (Fig. 2b, Treatment A). Despite the intense N_2_O production there was no evidence of atmosphere saturation inside the microcosm once the regressions performed to calculate N_2_O fluxes, as for CO_2_, also presented high correlation coefficient (minimum of 0.99). Microcosms N_2_O headspace concentrations reached 100 ppm after 90 min of incubation at the emission peak.

### Concluding remarks

• The analysis of the proposed method indicates that:the microcosms can be set up using materials available worldwide;
• the method was suitable to directly measure multiple trace gases fluxes, either produced or consumed during microcosm respiration, unlike the reference method;
• fluxes of CH_4_, N_2_O and CO_2_ were calculated from a wide range of gas headspace concentrations, demonstrating the method versatility;
• A series of fluxes measurements allows the determination of the analyte emission;
• Total emission is useful to calculate the biodegradability of materials or the effect of treatments on the microcosm respiration.
